# Aberrant expression of miR-33a-3p/IGF2 in postmenopausal osteoporosis patients and its role and mechanism in osteoporosis

**DOI:** 10.1186/s13018-023-03883-6

**Published:** 2023-07-06

**Authors:** Changxin Wang, Jianfei Shen, Wei Zhang, Xiaoyu Wang, Xiaohong Xu, Xianghui Lu, Dongbin Xu, Lan Yao

**Affiliations:** 1 Department of Orthopaedics, The Third Affiliated Hospital of Qiqihar Medical University, Qiqihar, 161000 China; 2 Nuclear Medicine Department, The Third Affiliated Hospital of Qiqihar Medical University, No. 27 Taishun Street, Tiefeng District, Qiqihar, 161000 China; 3 Endocrine Department, The Third Affiliated Hospital of Qiqihar Medical University, Qiqihar, 161000 China; 4 Department of Clinical Laboratory, The Third Affiliated Hospital of Qiqihar Medical University, Qiqihar, 161000 China; 5 Department of Gynaecology, The Third Affiliated Hospital of Qiqihar Medical University, Qiqihar, 161000 China; 6 Qiqihar Medical University, Qiqihar, 161000 China

**Keywords:** Postmenopausal osteoporosis, hBMSCs, miR-33a-3p/IGF2 axis

## Abstract

**Background:**

Postmenopausal osteoporosis (PMOP), the most frequent bone-related disease, is characterized by bone loss and fragile fractures, which is related to low bone density (BMD). This study aimed to illustrate the expression and mechanism of miR-33a-3p in osteoporosis.

**Methods:**

TargetScan and luciferase reporter assay were applied for verifying the relevance between miR-33a-3p and IGF2. Levels of miR-33a-3p, IGF2, Runx2, ALP and Osterix were checked using RT-qPCR and western blotting. hBMSCs proliferation, apoptosis and ALP activity were analyzed by MTT, flow cytometry (FCM) analysis and ALP detection kit, respectively. Moreover, the calcification of cells was assessed using Alizarin Red S staining. The average BMD was evaluated by dual-energy X-ray absorptiometry (DEXA) assay.

**Results:**

IGF2 was a target of miR-33a-3p. The level of miR-33a-3p was substantially higher and IGF2 expression was memorably lower in the serum of osteoporosis patients than that in healthy volunteers. Our results also pointed out that miR-33a-3p was reduced and IGF2 expression was enhanced during osteogenic differentiation. We concluded that miR-33a-3p negatively regulated the level of IGF2 in hBMSCs. Besides, miR-33a-3p mimic inhibited the osteogenic differentiation of hBMSCs via inhibiting the level of Runx2, ALP and Osterix and decreasing ALP activity. IGF2 plasmid dramatically reversed the influence of miR-33a-3p mimic on IGF2 expression, hBMSCs proliferation and apoptosis, and osteogenic differentiation of hBMSCs.

**Conclusion:**

miR-33a-3p affected osteogenic differentiation of hBMSCs by targeting IGF2, indicating a potential use of miR-33a-3p as plasma biomarker and therapeutic target for postmenopausal osteoporosis.

## Introduction

Osteoporosis is a systemic bone disease characterized by the reduction of bone mass and the degradation of the microstructure of bone tissue. The proportion of bone mineral composition and bone matrix keeps decreasing, resulting in the increase of bone fragility and the occurrence of fracture [1]. Osteoporosis can be divided into primary and secondary types. Primary osteoporosis can be divided into postmenopausal osteoporosis (type I), senile osteoporosis (type II) and idiopathic osteoporosis (including juvenile type) [2, 3]. Under normal circumstances, the whole body skeletal system is always in a dynamic balance between bone destruction and bone formation. The bone turnover rate in vivo is significantly accelerated, and the rate of bone absorption exceeds the rate of bone formation, resulting in the loss of bone mass, the decrease of bone density and the increase of bone fragility, leading to the occurrence of osteoporosis [4, 5]. Drug therapy is the main treatment method for osteoporosis [6–8]. In recent years, research on biomarkers of osteoporosis has received increasing attention [9, 10]. Therefore, the basic research on the pathogenesis of osteoporosis is of great significance to improve the level of clinical treatment. In particular, it is necessary to explore the biological functions and regulatory effects of osteoporosis-related genes, study the molecular mechanisms related to the occurrence and development of osteoporosis and find the therapeutic targets of osteoporosis.

Noncoding RNAs play critical roles in musculoskeletal conditions [11–14]. MicroRNAs (miRNAs) are a class of endogenous small RNAs with a length of about 20–24 nucleotides [15]. They have many important regulatory roles in cells, including organ formation, cell proliferation and apoptosis, and fat metabolism [16–18]. A large number of studies have shown that miRNAs are important regulators of bone metabolism [19]. Researchers have conducted a large number of studies on the target genes of bone metabolism related miRNA. For example, Hu et al. [20] suggested that miR-2861 plays an important role in promoting osteoblast differentiation by targeting histone deacetylase. Moreover, research from Jiang et al. [21] demonstrated that miR-204 inhibits osteogenic differentiation by regulating the expression of Runx2. MiR-33a-3p was evidenced to be involved in the progression of melanoma and human hepatocellular carcinoma. Studies have shown that miR-33a-3p inhibits cell migration and invasion by directly targeting human hepatocellular carcinoma PBX3 [22, 23]. It has been reported that the relative expression of miR-33a-3p was significantly decreased in the serum of osteoporosis after 3 months of tripatide treatment [24], which may be a promising therapeutic target. Thus, the role of miR-33a-3p in osteoporosis needs further study, and this is helpful to the pathological development of osteoporosis.

Through bioinformatics analysis, we found that IGF2 is a potential target gene for miR-33a-3p. Study has indicated that IGF2 was significantly down-regulated in the serums and bone tissues derived from osteoporotic patients, and it inhibited osteoclast differentiation [25]. IGF2 plays a crucial role in cell proliferation and osteogenic differentiation [26–28]. Therefore, we hypothesize that miR-33a-3p may affect the proliferation of MSCs and osteogenic differentiation by targeting IGF2.

Thus, our research was designed to illustrate the functions of miR-33a-3p in PMOP, and the role and underlying mechanism of miR-33a-3p in the proliferation and osteogenic differentiation of hBMSCs were investigated.

## Materials and methods

### Patient characteristics

Fifteen osteoporosis patients and healthy controls from the Third Affiliated Hospital of Qiqihar Medical University were included in the study. Written informed consent was obtained from all participants. Anthropometric characteristics, including age, BMI and T score of BMD, were recorded in the control and OP groups. The clinical data of osteoporosis patients and healthy volunteers are shown in Table 1. BMD was detected by Hologic 4500 bone densitometer and BMD was detected by a dual energy X-ray bone densitometer (DPX-NT, GE, WI, USA) according to WHO standards. This study was approved by the Ethics Committee of the Third Affiliated Hospital of Qiqihar Medical University.

**Table 1:** The clinical data of osteoporosis patients

Patient ID	Postmenopausal osteoporosis/Healthy control							
	Age (year)		BMI(kg/cm^2^)		BMD(g/cm^2^)		*T* value	
1	63.3	57	20.5	25.9	0.686	1.063	− 3.6	− 4.0
2	71.6	64	21.8	24.3	0.642	1.171	− 3.9	0.0
3	72.4	55.4	23.3	28.0	0.590	1.142	− 4.4	0.2
4	64.3	56	19.8	24.2	0.668	1.302	− 3.7	1.6
5	52.6	54.5	21.6	26.1	0.566	1.154	− 4.6	0.3
6	56.4	52.7	27.2	20.5	0.685	1.129	− 3.6	0.1
7	66.4	52.8	20.0	22.6	0.633	1.362	− 4.0	2.1
8	66.5	47.8	17.3	32.8	0.643	1.107	− 3.9	− 0.1
9	81.9	54.9	22.2	28.0	0.689	1.175	− 3.5	0.5
10	69.2	66.8	24.2	24.4	0.659	1.237	− 3.8	1.0
11	75	59.1	26.3	29.7	0.656	1.081	− 3.8	− 0.3
12	72.4	58.1	22.3	27.0	0.685	1.107	− 3.6	− 0.1
13	62.4	50.2	18.4	23.8	0.680	1.127	− 3.6	0.1
14	73	56.7	24.8	28.2	0.682	1.292	− 3.6	1.5
15	68.9	57.6	20.0	27.0	0.651	1.047	− 3.9	− 0.6

### Cell culture and stimulation

The hBMSCs were purchased from American Tissue Culture Collection (ATCC) and cultivated in DMEM (Gibco, NY, USA) containing 10% FBS and 1% penicillin/streptomycin (Procell) in a humidified incubator containing 5% CO_2_ at 37 °C. hBMSCs were induced in osteogenic induction medium containing 10% FBS, 5 mML glycerophosphate, 100 nM dexamethasone and 50 mg/ml ascorbic acid for 14 days. The induction medium was changed every three days during osteogenic differentiation.

### Alizarin red S staining

14 days after osteogenic induction, Alizarin red S staining was used to detect the calcification of hBMSCs. The hBMSCs were implanted in 96-well plates and treated with alizarin red S staining solution. The plates were gently washed twice with PBS to remove dead cells and fixed with 4% glutaraldehyde for 10 min at room temperature. After washing with distilled water for three times, the hBMSCs were stained at room temperature for 15 min. The hBMSCs were observed and photographed under an inverted microscope and the number of positive cells was counted with Image Pro Plus (IPP) software.

### ALP activity

14 days after osteogenic induction, hBMSCs were inoculated in 96 well plates and washed three times with PBS. Cells were fixed with 4% glutaraldehyde for 10 min at room temperature. Then, the cells were rinsed with distilled water for three times, and ALP activity was determined using ALP activity detection kit according to the product instructions.

### Dual-luciferase reporter assay

TargetScan was used to identify the relationship between miR-33a-3p and IGF2. The 3′-UTR of the IGF2 containing the miR-33a-3p binding site or mutated target site was amplified by RT-PCR and then cloned into a pmirGLO vector (Promega, USA) to generate the reporter vector IGF2-wild-type (IGF2-WT) or IGF2-mutated-type (IGF2-MUT). The results indicated that IGF2 was the potential target of miR-33a-3p. IGF2-WT or IGF2-MUT and miR-33a-3p mimic or mimic control were co-transfected into 293 T cells using Lipofectamine 3000, respectively, and incubated for 48 h. Then, the relative luciferase activity was detected by Dual-Luciferase^®^ Reporter Assay System (Promega, USA) following the protocol.

### RT-qPCR analysis

After treatment, the level of miR-33a-3p, IGF2, Runx2, ALP and Osterix were measured by RT-qPCR. The isolation of RNA from serum of osteoporosis patients and hBMSCs were obtained with the TRIpure Total RNA Extraction Reagent (ELK Biotechnology) based on the protocol. Then, the total RNA was reversed to cDNA following the instructions of PrimeScript RT Reagent Kit (TaKaRa, China) and RT-qPCR analysis was conducted using the SYBR PrimeScript RT-PCR Kit (TaKaRa) with ABI 7500 Real-Time PCR System (Agilent Technologies, USA). The thermocycling conditions were as following: Initial denaturation at 94 °C for 15 min; followed by 40 cycles at 94 °C for 15 s (denaturation), 60 °C for 15 s (annealing) and 72 °C for 15 s (extension).U6 for miRNA and GAPDH for mRNA were used as the internal controls. Target gene expressions were performed using the 2^−ΔΔCt^ method. Primer sequences for PCR were listed as following:miR-33a-3p forward, 5′ACACTCCAGCTGGGCAATGTTTCCACAGTG3′;reverse, 5′CTCAACTGGTGTCGTGGAGTCGGCAATTCAGTTGAGGTGATGCA3′;

IGF2 forward, 5′AGACCCTTTGCGGTGGAGA3′;reverse, 5′GGAAACATCTCGCTCGGACT3′;

U6 forward, 5′GCTTCGGCAGCACATATACTAAAAT3′;reverse, 5′CGCTTCACGAATTTGCGTGTCAT3′;

GAPDH forward, 5′CTTTGGTATCGTGGAAGGACTC3′;reverse, 5′GTAGAGGCAGGGATGATGTTCT3′;

Runx2 forward, 5′AGTCCCAACTTCCTGTGCTCC3′;reverse, 5′CGGTAACCACAGTCCCA TCTG3′.

ALP forward, 5′CTTGACTGTGGTTACTGCTGATCA3′;reverse, 5′GTATCCACCGAATGTGAAAACGT3′;

Osterix forward, 5′ACCAGGTCCAGGCAACAC3′;reverse, 5′-GCAAAGTCAGATGGGTAAGTAG-3′.

### Western blot assay

The hBMSCs were lysed using RIPA buffer (Beyotime) for 30 min on ice. Proteins were resolved by 10% SDS-PAGE and transferred onto PVDF membranes. The membranes were blocked with 5% skimmed milk for 2 h to avoid nonspecific binding and then cultivated with primary antibodies against IGF2 (cat. no. A2086, ABclonal; 1:1000 dilution) or β-actin (cat. no. TDY051; Beijing TDY Biotech CO., LTD.; 1:1000 dilution) at 4 °C overnight. After washing in TBST for three times, the membranes were cultivated with secondary antibodies for 2 h. The protein signals were assessed by ECL method following the instructions.

### Cell transfection

Mimic control, miR-33a-3p mimic, inhibitor control, miR-33a-3p inhibitor, control-plasmid, IGF2-plasmid were transfected into hBMSCs by Lipofectamine 2000 reagent (Invitrogen) for 48 h referring to the instructions. After 48 h transfection, RT-qPCR was used to detect miR-33a-3p expression. The RT-qPCR and western blot analysis were used to detect IGF2 expression.

### MTT assay

48 h after cell transfection with mimic control, miR-33a-3p mimic, miR-33a-3p mimic + control-plasmid or miR-33a-3p mimic + IGF2-plasmid, hBMSCs were implanted into 96-well plates and treated with 10 μl MTT solution and continuously incubated for additional 4 h. Then, the supernatant was discarded and 100 μl of DMSO was added to dissolve lysate without light. Finally, OD_570_ was measured by a microplate reader (BIOTEK, USA) following the protocol.

### Flow cytometry (FCM) assay

After transfected for 48 h, the hBMSCs were collected by centrifugation at 4 °C for 5 min. After that, the cells were washed twice with PBS. For cell apoptosis assay, hBMSCs were assessed using the Annexin-V/propidium iodide (PI) Apoptosis Detection Kit (Beyotime). The cells were gently mixed and were cultivated for 20 min at room temperature without light. Finally, apoptotic cells were checked using Flow cytometer (Beckman coulter) and analyzed using Kaluza Analysis software.

### Statistical analysis

Statistical analysis was conducted using GraphPad Prism 6.0 software. All results are expressed by mean ± SD from three independent experiments. The statistical significance among groups were determined by one-way ANOVA or Student’s t test. *P < 0.05 indicated as statistically significant*.*

## Results

### IGF2 acted as a direct target of miR-33a-3p

**Fig. 1:**
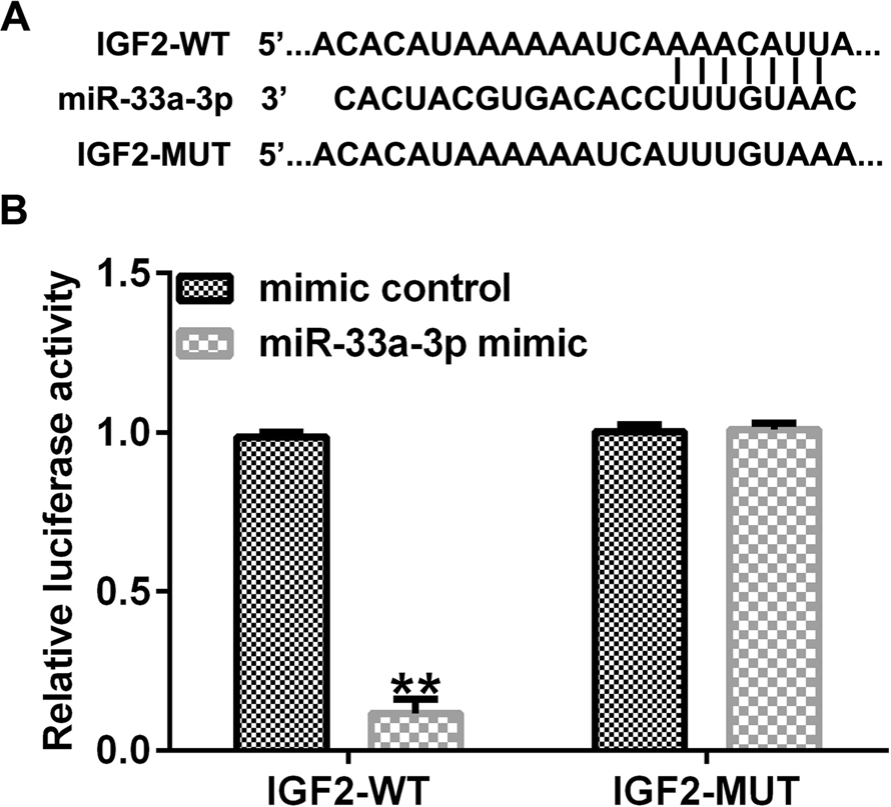
MiR-33a-3p directly targeted IGF2. **A** A schematic diagram of the forecasted miR-33a-3p binding site to IGF2. **B** Association between miR-33a-3p and IGF2 was confirmed by dual-luciferase reporter assay. **P < 0.01 versus mimic control

Firstly, we searched the downstream target gene of miR-33a-3p using TargetScan.org. We found that miR-33a-3p harbored a binding site for IGF2 (Fig. 1A). Dual-luciferase reporter gene system further suggested that IGF2-WT 3′-UTR with miR-33a-3p mimic substantially suppressed the relative luciferase activity, while the luciferase activity in IGF2-MUT group had no obvious change (Fig. 1B), revealing that miR-33a-3p directly targeted IGF2.

### miR-33a-3p and IGF2 expression in osteoporosis patients

**Fig. 2:**
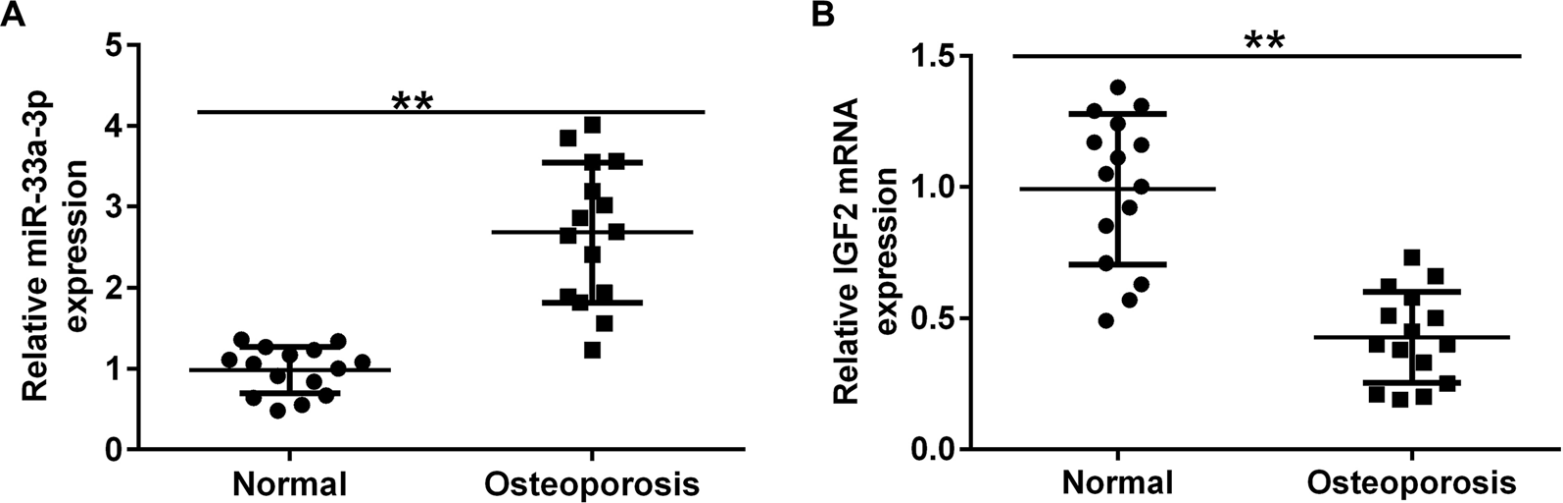
Expression of miR-33a-3p and IGF2 in the serum of osteoporosis patients. **A**–**B** Levels of miR-33a-3p and IGF2 in the serum of osteoporosis patients were checked by RT-qPCR assay.**P < 0.01

Besides, we evaluated the levels of miR-33a-3p and IGF2 in the serum of osteoporosis patients. As displayed in Fig. 2A,B, miR-33a-3p was up-regulated and IGF2 was down-expressed in the serum of osteoporosis patients, as exposed to the healthy control group.

### Osteogenic differentiation induction reduced miR-33a-3p expression and enhanced IGF2 levels

**Fig. 3:**
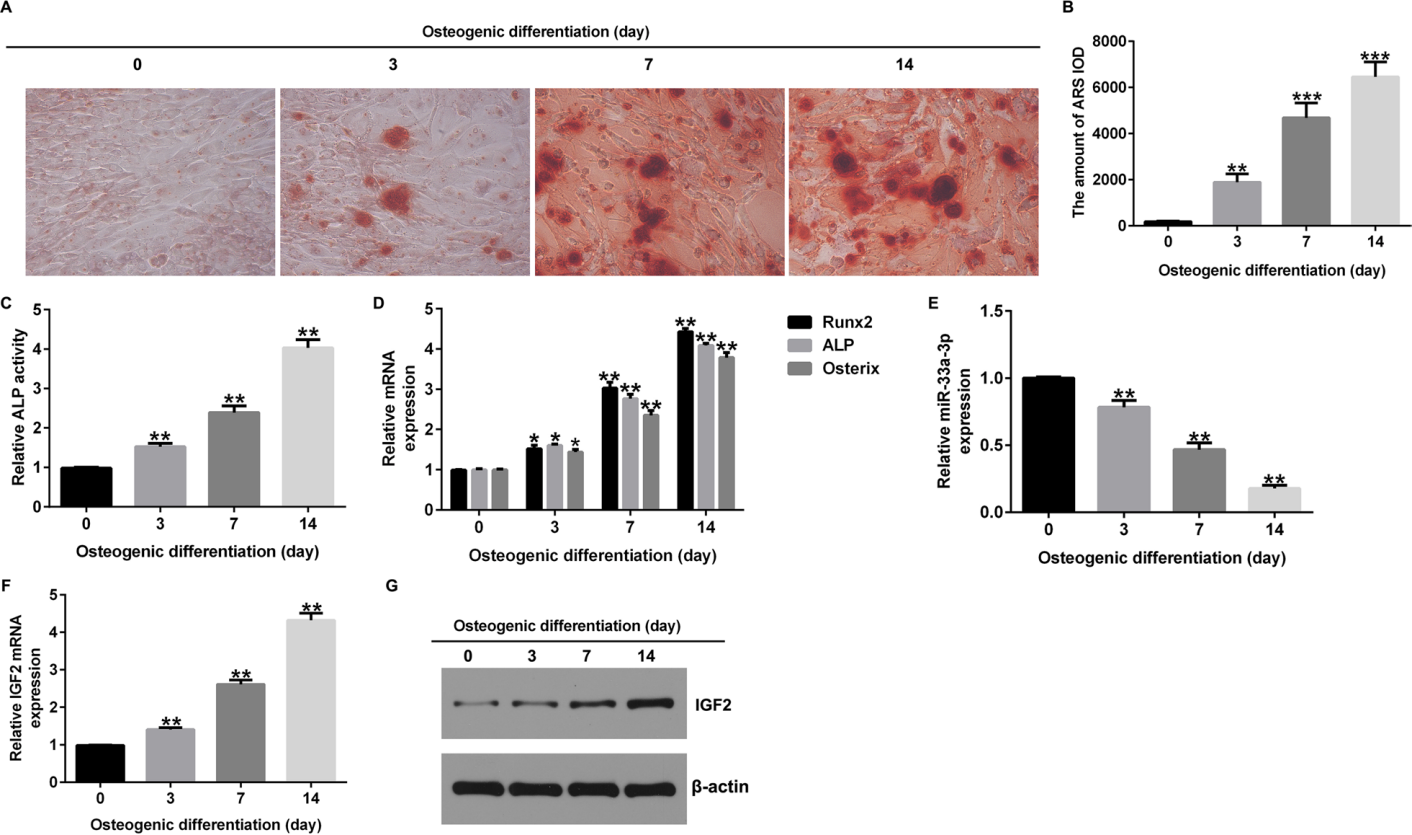
Expression of miR-33a-3p and IGF2 in osteogenic differentiation induced hBMSCs. **A** Alizarin red S was applied for detecting calcified cells. **B** Quantification of calcified cells. **C** Detection of ALP activity. **D** RT-qPCR analysis of Runx2, ALP and Osterix mRNA levels. **E**–**G** The expression of miR-33a-3p and IGF2 was assessed by western blot analysis or RT-qPCR analysis. *P < 0.05, **P < 0.01, ***P < 0.001 versus Control

After osteogenic differentiation induction for 14 days, Alizarin red S staining was applied for evaluating the calcification of cells. Our data suggested that the calcification of cells became increasing serious over time (Fig. 3A, B). In clinical practice, serum ALP activity is often used as a clinical auxiliary diagnostic index for skeletal diseases. Then, we detected the ALP activity using ALP activity kit, and our data revealed that the ALP activity in osteogenic differentiation induced cells was enhanced, compared to the control group (Fig. 3C). Results from RT-qPCR suggested that osteoblast makers, including Runx2, ALP and Osterix, were markedly enhanced by osteogenic differentiation induction (Fig. 3C). Furthermore, we determined the levels of miR-33a-3p and IGF2 in osteogenic differentiation inducted cells. As displayed in Fig. 3E–G, miR-33a-3p was down-expressed and IGF2 was up-regulated in osteogenic differentiation inducted cells, suggesting that miR-33a-3p and IGF2 participated in osteogenic differentiation of hBMSCs.

### MiR-33a-3p negatively regulated IGF2 expression in hBMSCs

**Fig. 4:**
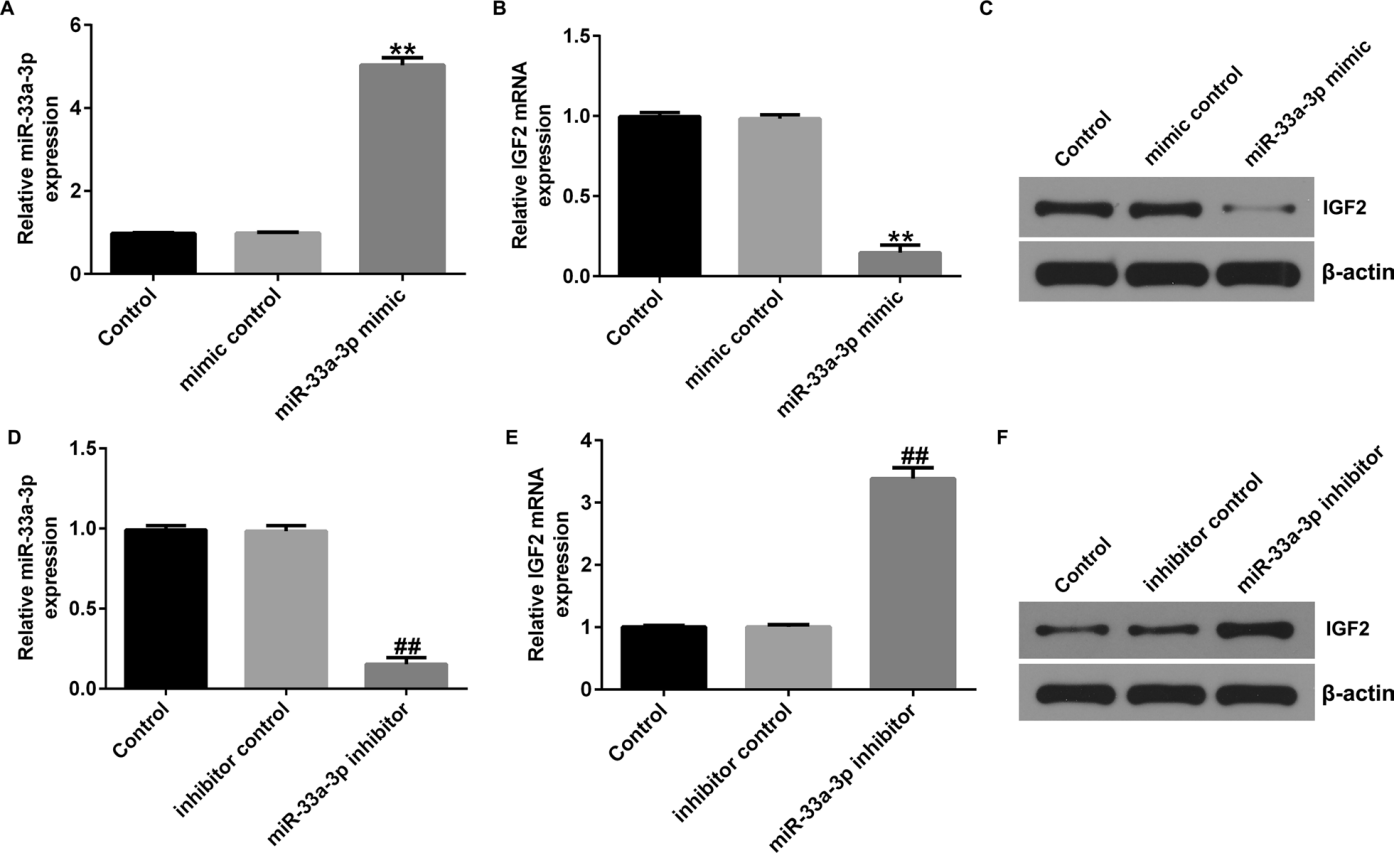
Effects of miR-33a-3p on IGF2 levels in hBMSCs. hBMSCs were transfected with mimic control, miR-33a-3p mimic, inhibitor control or miR-33a-3p inhibitor for 48 h. **A** Detection of miR-33a-3p levels using RT-qPCR. **B**–**C** Expressions of IGF2 were assessed by RT-qPCR and western blot analysis. RT-qPCR analysis of miR-33a-3p (**D**) and IGF2 (**E**) mRNA levels in miR-33a-3p inhibitor or inhibitor control transfected cells. **F** Western blot analysis of miR-33a-3p and IGF2. **P < 0.01 versus Mimic control; ##*P* < 0.01 versus Inhibitor control

To further assess the relevance between miR-33a-3p and IGF2 in hBMSCs, hBMSCs were transfected with mimic control, miR-33a-3p mimic, inhibitor control or miR-33a-3p inhibitor for 48 h. The transfection efficiency was checked by RT-qPCR. We observed that miR-33a-3p mimic dramatically promoted miR-33a-3p expression, and suppressed IGF2 level, in comparison with mimic control group (Fig. 4A–C). Moreover, silencing of miR-33a-3p signally reduced miR-33a-3p level and enhanced IGF2 expression in hBMSCs (Fig. 4D–F). Based on these findings, we concluded that miR-33a-3p negatively regulated the level of IGF2 in hBMSCs.

### IGF2 plasmid significantly reversed the effects of miR-33a-3p mimic on IGF2 expression

**Fig. 5:**
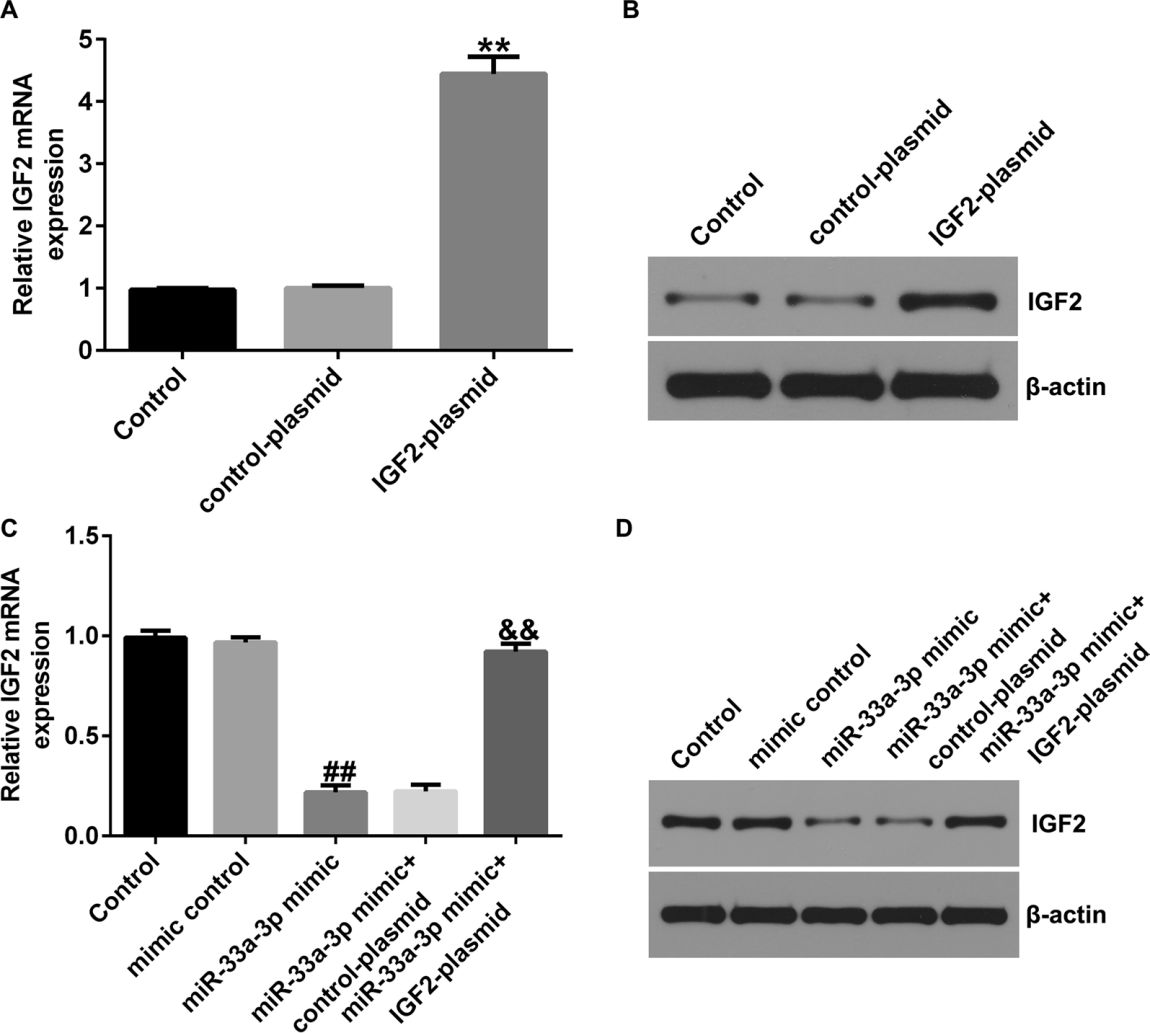
Effects of IGF2-plasmid or miR-33a-3p mimic on IGF2 levels in hBMSCs. hBMSCs were transfected with mimic control, miR-33a-3p mimic, control-plasmid, IGF2-plasmid for 48 h. **A**–**B** Determination of IGF2 level in control-plasmid or IGF2-plasmid transfected cells by RT-qPCR and western blot assay. **C**–**D** Expressions of IGF2 were assessed by RT-qPCR and western blot analysis. **P < 0.01 versus Control-plasmid; ##*P* < 0.01 versus Mimic control; &&*P* < 0.01 versus miR-33a-3p mimic + control-plasmid

Besides, control-plasmid, IGF2-plasmid, mimic control, miR-33a-3p mimic, miR-33a-3p mimic + control-plasmid or miR-33a-3p mimic + IGF2-plasmid was transfected into hBMSCs for 48 h. As presented in Fig. 5A, B, the level of IGF2 was remarkably higher in IGF2-plasmid transfected hBMSCs than that in control-plasmid group. Moreover, results from RT-qPCR and western blot analysis suggested that miR-33a-3p mimic remarkably inhibited IGF2 level, as exposed to mimic control group (Fig. 5C and D), while this inhibition was reversed by IGF2-plasmid. These data revealed that IGF2 plasmid significantly reversed the inhibitory effects of miR-33a-3p mimic on IGF2 expression in hBMSCs.

### IGF2-plasmid reversed the roles of miR-33a-3p mimic in osteogenic differentiation induced hBMSCs

**Fig. 6:**
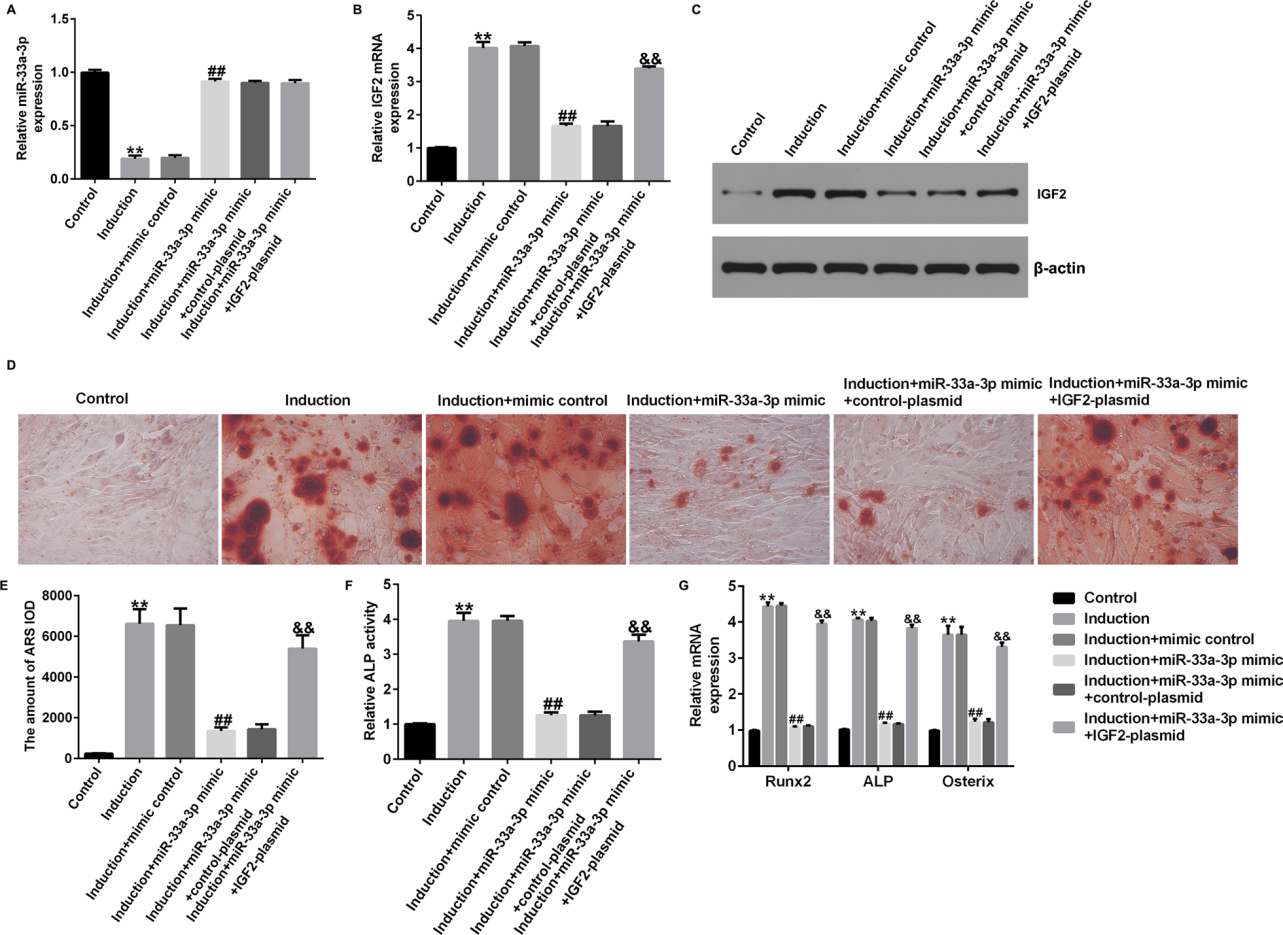
Effects of miR-33a-3p mimic or IGF2-plasmid on hBMSCs calcification and ALP activity. hBMSCs were transfected with mimic control, miR-33a-3p mimic, control-plasmid, IGF2-plasmid for 48 h. RT-qPCR analysis and western blot assay of miR-33a-3p (**A**) and IGF2 (**B**–**C**) expression. **D** Calcified cells were determined by Alizarin Red S. **E** Quantification of calcified cells. **F** Evaluation of ALP activity. **G** mRNA levels of Runx2, ALP and Osterix were detected by RT-qPCR analysis. **P < 0.01 versus Control; ##*P* < 0.01 versus Induction + mimic control; &&*P* < 0.01 versus Induction + miR-33a-3p mimic + control-plasmid

To further illustrate the mechanism of miR-33a-3p and IGF2 in osteoporosis, hBMSCs were transfected with mimic control, miR-33a-3p mimic, miR-33a-3p mimic + control-plasmid or miR-33a-3p mimic + IGF2-plasmid for 48 h and then stimulated with osteogenic induction medium for 14 days. We observed suppressed miR-33a-3p expression (Fig. 6A), enhanced IGF2 level (Fig. 6B, C), calcification cells (Fig. 6D and E), ALP activity (Fig. 6F) and Runx2, ALP and Osterix mRNA levels (Fig. 6G) in osteogenic differentiation induction group cells, while we observed the opposite findings in miR-33a-3p mimic transfected cells. However, these results caused by miR-33a-3p mimic were successfully abolished by IGF2-plasmid, suggesting that IGF2-plasmid reversed the functions of miR-33a-3p mimic in osteogenic differentiation induced hBMSCs.

### MiR-33a-3p inhibited hBMSCs proliferation and induced hBMSCs apoptosis by down regulating IGF2

**Fig. 7:**
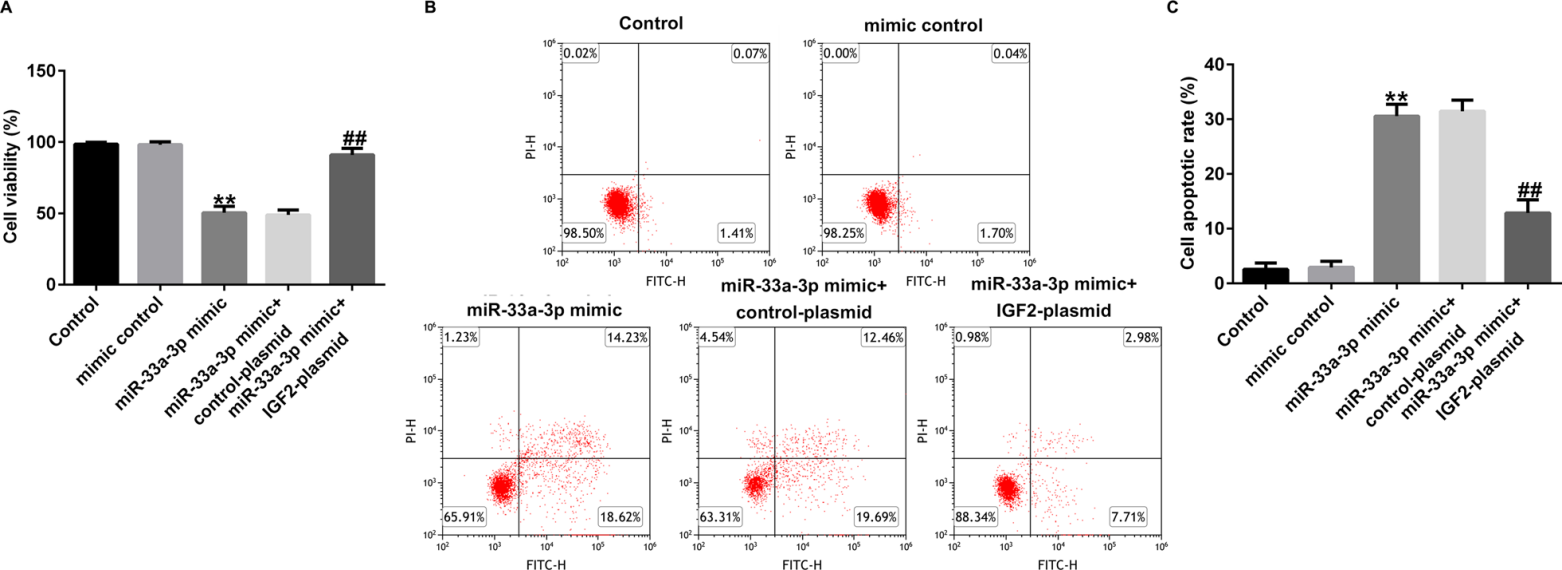
Effects of miR-33a-3p mimic or IGF2-plasmid on hBMSCs viability and apoptosis. hBMSCs were transfected with mimic control, miR-33a-3p mimic, control-plasmid, IGF2-plasmid for 48 h. **A** MTT assay of cell viability. **B** Cell apoptosis was detected by Flow cytometry assay. **C** Quantification of apoptotic cells. **P < 0.01 versus mimic control; ##*P* < 0.01 versus miR-33a-3p mimic + control-plasmid

Moreover, we uncovered the roles of miR-33a-3p on hBMSCs proliferation and apoptosis. hBMSCs were transfected with mimic control, miR-33a-3p mimic, miR-33a-3p mimic + control-plasmid or miR-33a-3p mimic + IGF2-plasmid for 48 h. Results from MTT and flow cytometry analysis demonstrated that miR-33a-3p mimic remarkably suppressed cells viability (Fig. 7A), and stimulated more apoptotic hBMSCs (Fig. 7B and C), while these findings were reversed in IGF2-plasmid transfected hBMSCs. Our data indicated that IGF2-plasmid relieved miR-33a-3p-induced hBMSCs viability reduction and apoptosis increase. Taken these data together, miR-33a-3p/IGF2 was evidenced to be involved in the progression of osteoporosis.

## Discussion

PMOP, the most frequent metabolic bone diseases, affected the postmenopausal women's health all over the world. Osteoporosis is a bone disease characterized by impaired bone strength, reduction of bone mineral content and bone matrix composition in equal proportion, leading to an increased risk of fracture [29]. At present, calcitonin [30], hormone replacement therapy [31] and zoledronic acid [32] are the main methods of PMOP treatment. Nevertheless, detailed mechanism and strategies of treatment for PMOP remain insufficient.

Increasing evidence has revealed that miRNAs are involved in regulating osteoporosis pathogenesis and could be acted as latent therapeutic targets. Zhao et al. [33] revealed that silencing of miR-483-5p alleviates PMOP through PI3K/AKT pathway. Report from Yu et al. suggested miR-16-5p regulates PMOP by directly targeting VEGFA. MiR-33a-3p was evidenced to play vital roles in multiple diseases, including melanoma [34]. Besides, miR-33a-3p was significantly reduced in the serum of osteoporosis after 3 months of tripatide treatment [24]. However, the specific function of miR-33a-3p in PMOP needs further study. Therefore, in this report, we focused on illustrating the roles of miR-33a-3p in PMOP. Firstly, we searched the related gene of miR-33a-3p and found that miR-33a-3p sponging to IGF2. Besides, we determined the levels of miR-33a-3p and IGF2 in the serum of osteoporosis patients and explored the potential associations with BMD. Our data suggested that miR-33a-3p was over-expressed and IGF2 was down-regulated in the serum of osteoporosis patients, as exposed to control group. In our research, the serum miR-33a-3p levels had negative correlations in T score of BMD, while the serum IGF2 expressions displayed the opposite results. Furthermore, obvious negative correlations were presented between miR-33a-3p and IGF2 in PMOP. Therefore, to clarify the expression difference of miR-33a-3p and IGF2 in the osteogenic differentiation of hBMSCs has guiding significance for the development of osteoporosis.

Recently, miR-33a-3p has been shown to be involved in calcium deposition, but its mechanism for cellular calcification is unclear. We observed that the calcification of cells became increasing serious over time. During the differentiation of hBMSCs into osteoblasts, we observed an increase in the following bone markers: ALP, Runx2 and Osterix. All of these proteins indicate the possible presence of active bone formation [35–37]. Our data also revealed that the ALP activity in osteogenic differentiation induced cells was enhanced, compared to control group cells. MiR-33a-3p was down-expressed and IGF2 was up-regulated in osteogenic differentiation inducted cells, demonstrating that miR-33a-3p and IGF2 were participated in osteogenic differentiation of hBMSCs. To further explain the relevance between miR-33a-3p and IGF2 in hBMSCs, hBMSCs were transfected with mimic control, miR-33a-3p mimic, inhibitor control, miR-33a-3p inhibitor, control-plasmid or IGF2-plasmid. Our data revealed that miR-33a-3p negatively regulated the level of IGF2 in hBMSCs, and IGF2 plasmid significantly reversed the effects of miR-33a-3p mimic on IGF2 expression. Our findings above revealed that miR-33a-3p and IGF2 were associated with PMOP progression.

To further explain the mechanism of miR-33a-3p and IGF2 in osteogenic differentiation of hBMSCs, hBMSCs were stimulated with osteogenic induction medium, and transfected with mimic control, miR-33a-3p mimic, control-plasmid or IGF2-plasmid. Our data suggested that IGF2-plasmid reversed the functions of miR-33a-3p mimic in induced hBMSCs, as confirmed by suppressed miR-33a-3p expression, enhanced IGF2 level, stimulated more calcification cells, increased Runx2, ALP and Osterix mRNA levels and ALP activity, our data suggested that IGF2-plasmid reversed the roles of miR-33a-3p mimic in induced hBMSCs. A large number of researches have demonstrated that miRNAs were associated with biological functions, including growth, apoptosis and metastasis in PMOP. For example, previous report has suggested that miR-491-3p is down-regulated in PMOP and affects growth, differentiation and apoptosis of hFOB1.19 cells through targeting CTSS [38]. Then, we conducted functional assays of miR-33a-3p mimic or IGF2-plasmid to explain whether it mediated PMOP. Our data indicated that IGF2-plasmid relieved miR-33a-3p-induced hBMSCs viability and apoptosis.

Based on the above investigations, this study suggested that miR-33a-3p silencing plays a protective role in PMOP progression via promoting the osteogenic differentiation of hBMSCs and suppressing apoptosis by targeting IGF2. Therefore, miR-33a-3p may be regarded as a promising target for PMOP treatment. Additional in vivo experiments are needed to verify the detailed mechanism of miR-33a-3p in PMOP progression.

## Data Availability

The datasets used and/or analyzed during the current study are available from the corresponding author on reasonable request.
