# Correction: Aberrant expression of miR-33a-3p/IGF2 in postmenopausal osteoporosis patients and its role and mechanism in osteoporosis

**DOI:** 10.1186/s13018-024-05042-x

**Published:** 2024-09-12

**Authors:** Changxin Wang, Jianfei Shen, Wei Zhang, Xiaoyu Wang, Xiaohong Xu, Xianghui Lu, Dongbin Xu, Lan Yao

**Affiliations:** 1 Department of Orthopaedics, The Third Affiliated Hospital of Qiqihar Medical University, Qiqihar, 161000 China; 2 Nuclear Medicine Department, The Third Affiliated Hospital of Qiqihar Medical University, No. 27 Taishun Street, Tiefeng Distric, Qiqihar, 161000 China; 3 Endocrine Department, The Third Affiliated Hospital of Qiqihar Medical University, Qiqihar, 161000 China; 4 Department of Clinical Laboratory, The Third Affiliated Hospital of Qiqihar Medical University, Qiqihar, 161000 China; 5 Department of Gynaecology, The Third Affiliated Hospital of Qiqihar Medical University, Qiqihar, 161000 China; 6 Qiqihar Medical University, Qiqihar, 161000 China

**Correction: Journal of Orthopaedic Surgery and Research (2023) 18: 487**

10.1186/s13018-023-03883-6

In the sentence beginning ‘Fifteen osteoporosis patients...’ in this article, the incorrect text dual-energy X-ray bone densitometer (DEXA) should have read ‘BMD was detected by a dual energy X-ray bone densitometer (DPX-NT, GE, WI, USA)’.

The updated sentence is given below and the changes have been highlighted in **bold typeface**.

## Materials and methods

### Patient characteristics

Fifteen osteoporosis patients and healthy controls from the Third Affiliated Hospital of Qiqihar Medical University were included in the study. Written informed consent was obtained from all participants. Anthropometric characteristics, including age, BMI, and T-score of BMD, were recorded in the control and OP groups. The clinical data of osteoporosis patients and healthy volunteers were shown in Table 1. **BMD was detected by a dual energy X-ray bone densitometer (DPX-NT**,** GE**,** WI**,** USA)** according to WHO standards. This study was approved by the Ethics Committee of the Third Affiliated Hospital of Qiqihar Medical University.

